# Plasma concentration of *Propionibacterium acnes* antibodies and prostate cancer risk: results from an Australian population-based case–control study

**DOI:** 10.1038/sj.bjc.6605757

**Published:** 2010-07-06

**Authors:** G Severi, B A Shannon, H N Hoang, L Baglietto, D R English, J L Hopper, J Pedersen, M C Southey, R Sinclair, R J Cohen, G G Giles

**Affiliations:** 1 Cancer Epidemiology Centre, The Cancer Council of Victoria, Melbourne, Victoria 3053, Australia; 2 Centre for Molecular, Environmental, Genetic and Analytic Epidemiology, The University of Melbourne, Melbourne, Victoria 3052, Australia; 3 Tissugen Pty Ltd., Perth, Western Australia 6009, Australia; 4 School of Pathology and Laboratory Medicine, University of Western Australia, Perth, Western Australia 6009, Australia; 5 Tissupath Pty Ltd., Melbourne, Victoria 3122, Australia; 6 Genetic Epidemiology Laboratory, Department of Pathology, The University of Melbourne, Melbourne, Victoria 3052, Australia; 7 Department of Dermatology, St Vincent's Hospital, Melbourne, Victoria 3065, Australia; 8 Uropath Pty Ltd., Perth, Western Australia 6009, Australia

**Keywords:** prostate cancer, risk factor, acne, *P. acnes*, case–control study

## Abstract

**Background:**

Recent studies in prostatic tissue suggest that *Propionibacterium acnes* (*P. acnes*), a bacterium associated with acne that normally lives on the skin, is the most prevalent bacterium in the prostate and in men with benign prostatic hyperplasia. Its prevalence is higher in samples from patients subsequently diagnosed with prostate cancer. The aim of our study was to test whether circulating levels of *P. acnes* antibodies are associated with prostate cancer risk and tumour characteristics using plasma samples from a population-based case–control study.

**Methods:**

We measured plasma concentration of *P. acnes* antibodies for 809 cases and 584 controls using a recently developed ELISA assay. We compared antibody titres between cases and controls using unconditional logistic regression adjusted for batch and variables associated with the study design (i.e., age, year of selection and centre). The primary analysis included *P. acnes* titres in the model as a dichotomous variable using the median value for controls as the cut-off value.

**Results:**

*P. acnes* antibody titres for both cases and controls ranged from 1 : 16 (i.e., low concentration) to 1 : 65 536 (i.e., high concentration; median value=1 : 1024). The odds ratio for prostate cancer associated with titres at or above the median value was 0.73 (95% CI 0.58–0.91, *P*=0.005). The association appeared to be particularly strong for advanced prostate cancer (AJCC Stage grouping III–IV) for which the odds ratio was 0.59 (95% CI 0.43–0.81, *P*=0.001) but there was insufficient evidence that the association differed by tumour stage (*p heterogeneity*=0.07).

**Conclusion:**

These results need to be confirmed in prospective studies but they are consistent with the hypothesis that *P. acnes* has a role in prostate cancer.

Studies investigating a possible association between acne and prostate cancer risk have reported inconsistent results. These include a decreased risk associated with facial acne scarring in our Risk Factors for Prostate Cancer Study (RFPCS) (Giles *et al*, 2003), no significant associations in other two studies (Lightfoot *et al*, 2004; Galobardes *et al*, 2005), and an increased risk associated with long-term use of tetracyclines that was assumed to indicate treatment of severe acne (Sutcliffe *et al*, 2007). Most of these studies (Giles *et al*, 2003; Lightfoot *et al*, 2004; Galobardes *et al*, 2005) were investigating acne as a marker of androgen activity in puberty, under the hypothesis that higher androgen levels might predispose to both acne and development of prostate cancer.

Subsequent research has indicated another possible connection between acne and prostate cancer. Recent reports on *Propionibacterium acnes* (*P. acnes*), a bacterium associated with acne that normally lives on the skin and thrives in blocked follicles, suggest that this bacterium is prevalent in the prostate, it is associated with acute and chronic prostatic inflammation, and it might have a role in prostate carcinogenesis (Cohen *et al*, 2005; Alexeyev *et al*, 2006). Our group (RJC and BAS) cultured *P. acnes* from one-third of a series of radical prostatectomy specimens and found that *P. acnes*-positive specimens were more likely to contain foci of acute or chronic inflammation than *P. acnes*-negative specimens (Cohen *et al*, 2005). Consistent with these findings Alexeyev *et al* (2006) investigating bacterial DNA in prostatic tissue from patients with benign prostatic hyperplasia found that *P. acnes* was the most prevalent bacterium and that its prevalence was higher in samples from patients subsequently diagnosed with prostate cancer. These observations led to hypothesise that *P. acnes* infection in the prostate gland is positively associated with prostate cancer risk, with acne considered as a marker of increased immune sensitivity to *P. acnes* infection.

The aim of our study was to test whether plasma concentration of *P. acnes* antibodies, measured by an ELISA assay we recently developed (Shannon *et al*, 2008), was associated with prostate cancer risk. We tested this hypothesis using the RFPCS, a population-based case–control study. Although the majority of studies discussed above have suggested positive associations between prostate cancer and either acne in puberty or *P. acnes* infection in the prostate gland, our RFPCS cohort previously showed an inverse association between facial scarring and prostate cancer risk (Giles *et al*, 2003), therefore a secondary aim of this study was to investigate this apparently conflicting result.

## Materials and methods

### Study design and subjects

The RFPCS study was conducted in 1994–1998 in Melbourne and Perth, Australia. Details of this study were published previously (Giles *et al*, 2001; Severi *et al*, 2003). Cases were men aged less than 70 years at diagnosis with histologically confirmed adenocarcinoma of the prostate and notified to the population-based Cancer Registries in the two States during the period between 1994 and 1997. Tumours were staged according to the TNM system and categorised into American Joint Committee on Cancer stage groupings (I–IV) (AJCC, 2002). Cases were classified into two groups according to tumour differentiation: moderate-grade cases (i.e., Gleason score 5–7) and high-grade cases (i.e., Gleason score 8–10). Men with well-differentiated tumours or Gleason score less than 5 were excluded. Controls were randomly selected from the state electoral rolls and frequency matched to cases by 5-year age group and city of residence at the time of selection in a ratio of one control per case. Face to face interviews were conducted usually at the men's home using questionnaires to capture various exposures including demographic factors, medical history, anthropometry, radiation exposures, alcohol consumption, smoking, growth and development, medical and surgical procedures related to the reproductive organs, diet captured using a detailed food frequency questionnaire, lifetime history of sexual activity, and family history of prostate cancer. The interview also included questions about acne in adolescence. The interviewer was asked to report ‘obvious’ acne scarring or ‘uncertain’ acne scarring (e.g., beard). About mid study, additional funding was obtained to collect blood samples from subjects mainly to extract DNA for genetic association studies and measure levels of markers in circulation. The total number of samples available for this study was 809 for cases and 584 for controls.

### Measure of *P. acnes* antibodies

Anti-*P. acnes* antibody titres were determined at Tissugen Pty Ltd. (Perth, Western Australia, Australia) by researchers blinded to the case–control status of the plasma samples. The assay was performed as previously described (Shannon *et al*, 2008), except that incubation with primary and secondary antibodies (2 h each, with agitation) and the colour development step (30 min) were carried out at 37°C. Briefly, the cell wall-associated/secreted surface proteins harvested from a cultured prostatic *P. acnes* isolate, (type II) were purified, resolubilised to 1.5 *μ*g ml^–1^ in 0.05 M carbonate buffer (pH 9.6) and coated overnight onto 96-well ELISA plates. Each plate included no-antigen and no-plasma blanks plus the positive control plasma sample (diluted to 1 : 64). Plasma samples were analysed in duplicate using two-fold serial dilutions. Absorbance readings were multiplied by a standardisation factor (the amount required to adjust the positive control to its mean value of 0.957) to allow accurate comparison of samples from different runs. The endpoint antibody titre was then determined as the highest serum dilution giving an absorbance reading of 0.100 or greater. The coefficient of intra-assay variation was 5.7% (as calculated from 18 duplicates of positive control in the same ELISA run) and the inter-assay variation was 6.3% (as calculated from 18 different ELISA runs of the positive control).

Plasma samples from 69 men were divided into two aliquots to check the replicability of the measures. The laboratory was blind to the duplicates that were included at random among the samples used for the study. As a measure of replicability we used the intra-class correlation coefficient, which is the proportion of the total variance due to variation between persons, where the total variance included a component due to between persons and between measures.

### Statistical methods

The primary hypothesis of association between *P. acnes* antibody titres and prostate cancer risk was tested using unconditional logistic regression with the case–control status as a dependent variable (Breslow and Day, 1980). *P. acnes* titres were dichotomised using the median value for controls (i.e., 1 : 1024) and included as independent variable in the logistic model. Odds ratio (OR) estimates and their 95% confidence intervals (CI) were derived under the likelihood theory. We performed secondary analyses using the tertiles of the distribution of *P. acnes* titres in controls and using the original continuous variable. We log_2_ transformed the continuous variable before inclusion in the model so that the odds ratio would represent the relative difference in risk associated with a doubling of the titres. We fitted models adjusted for self-reported history of acne during adolescence or facial acne scarring to investigate whether this would affect the association between *P. acnes* titres and prostate cancer risk. We also tested the interaction between *P. acnes* titres and prostate cancer risk and self-reported history of acne in adolescence, facial acne scarring and reference age (age at diagnosis for cases and age at selection for controls). Odd ratios by tumour stage (stage I–II and stage III–IV) and grade (moderate-grade and high-grade) were estimated using polytomous logistic regression models. All regression analyses were adjusted for batch and for variables associated with the original study design: reference age (<55, 55–59, 60–69), study centre (Melbourne and Perth), selection year (1994, 1995, 1996, and 1997). Further adjustment for family history of prostate cancer (i.e., number of first-degree relatives affected), country of birth (Australia *vs* others), and self-reported history of benign prostatic hyperplasia did not materially change the estimates. We tested whether adult *P. acnes* antibody titres were associated with self-reported acne during adolescence or facial acne scarring by applying linear regression to the logarithm of the inverse of the titres for controls using history of acne or facial scarring as predictors. We tested for trend in the odds ratios by comparing the likelihoods of two models with and without a pseudo-continuous variable that was constructed first by log_2_ transforming the original variable and then by assigning each man in a specific tertile, the corresponding median value for that tertile. We tested hypotheses using the likelihood ratio test. All tests were two-sided and nominal statistical significance was based on *P*<0.05. All statistical analyses were performed using Stata/SE 10 (Stata Corporation, College Station, Texas, USA).

## Results

We measured *P. acnes* antibody titres for a total of 809 cases and 584 controls whose characteristics are described in Table 1. There was little variation between the duplicates as indicated by the high intra-class correlation coefficient (0.94; 95% CI 0.91–0.97). *P. acnes* antibody titres measured for RFPCS participants ranged from 1 : 16 (i.e., low concentration) to 1 : 65 536 (i.e., high concentration) and the median value was 1 : 1024. The mean log_2_ titres for controls with and without a history of acne were 10.6 and 9.8, respectively. The analysis of *P. acnes* titres for controls showed that after adjusting for the study variables titres for men reporting a history of acne in adolescence were 85% higher than titres for those not reporting a history of acne (*P*=0.003). We found similar results for facial acne scarring (data not shown).

**Figure 1:**
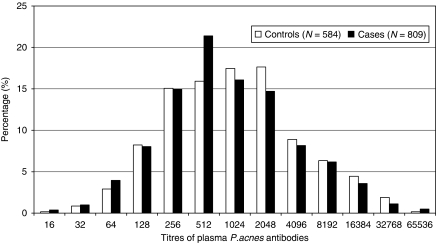
Distribution of *P.acnes* antibody titres for prostate cancer cases and controls from the Australian Risk Factors for Prostate Cancer Study.

The distribution of *P. acnes* antibody titres is presented separately for cases and controls in Figure 1. The median and range of the titres were the same for cases and controls but samples with high titres (e.g., 1 : 2048 or more) appeared to be more prevalent for controls than for cases. The mean log_2_ titres for controls and cases were 10 and 9.7, respectively. When we fitted a logistic regression model to the titres split in two groups using the median as cut-off point (1 : 1024), the odds ratio associated with titres equal to or above the median compared with titres below the median was 0.73 (95% CI 0.58–0.91) (Table 2). The odds ratio was 0.59 (95% CI 0.43–0.81) for advanced prostate cancer (stage III–IV) and 0.79 (95% CI 0.62–1.01) for early-stage prostate cancer (stage I–II), but there was insufficient evidence to conclude that the association differs by tumour stage (p-heterogeneity=0.07). The odds ratios for moderate-grade prostate cancer were very similar to the odds ratios for high-grade prostate cancer (p-heterogeneity=0.8). The association between *P. acnes* antibody titres was confirmed when we fitted the logistic regression including the titres as continuous variables. The odd ratio for doubling the antibody titres was 0.94 (95% CI 0.90–0.99). In the analysis by tertiles of the distribution of *P. acnes* titres in controls, the odds ratios associated with the second and third tertiles compared with the first tertile were 1.00 (95% CI 0.76–1.34) and 0.80 (95% CI 0.60–1.06), respectively (test for linear trend, *P*=0.06).

The odds ratios for *P. acnes* titres did not materially change after the inclusion of self-reported history of acne or facial acne scarring in the models (data not shown), indicating that the association between *P. acnes* antibody titres and prostate cancer risk is independent of self-reported history of acne during adolescence or facial acne scarring.

For self-reported history of acne during adolescence the odds ratio for overall prostate cancer was 0.93 (95% CI 0.70–1.22). For facial acne scarring, the odds ratios for overall prostate cancer was 0.66 (95% CI 0.37–1.17) for the obvious compared with the not obvious. We found no statistically significant evidence of interactions between *P. acnes* antibody titres and self-reported history of acne during adolescence or *P. acnes* antibody titres and facial acne scarring after removing men with uncertain acne scarring.

## Discussion

The findings from our case–control study suggest that higher concentrations of circulating *P. acnes* antibodies measured in adulthood are associated with decreased risk of prostate cancer, especially for advanced prostate cancer. The strengths of this study include the excellent repeatability of the plasma measures, the population-based design, and the relatively large sample size. One limitation is the lack of specificity of the *P. acnes* antibodies for subtypes prevalent in the prostate. Although the ELISA was developed from isolates cultured from the prostate, immunoblots showed that antigens were conserved among other types of *P. acnes* and thus our assay could not differentiate between immune responses raised against *P. acnes* infection in the prostate and *P. acnes* infection in the skin or in other organs. In our retrospective study blood samples for cases were collected after diagnosis and we cannot rule out that the antibody titres in our prostate cancer cases were lowered by the tumour itself or by androgen-deprivation therapy. However, this is unlikely because cancer tends to upregulate the antibody-mediated immune response (Filella *et al*, 2000; Johansson *et al*, 2008) and evidence suggests that androgen deprivation therapy may increase, rather than decrease, immune function and antibody production (Aragon-Ching *et al*, 2007).

Since *P. acnes* induces a strong cell-mediated T-helper type 1 (Th1) immune response (Mouser *et al*, 2003; Namazi, 2007) a more likely explanation of our findings is that higher antibody titres are an indirect marker of increased cell-mediated immunity caused by *P. acnes* infection and that this increased immunity protects from prostate cancer.

Persistent exposure to Th1 cytokines through chronic infection can result in systemic Th1 polarisation of the immune system (Romagnani, 2004), therefore, people with a history of acne or *P. acnes*-related infection of the prostate gland could be predisposed to develop a strong systemic Th1-type immune response. Th1 immune responses are detrimental to the establishment and progression of tumours, which typically secrete factors to suppress Th1 and promote T-helper type 2 (humoral or antibody-mediated) immunity (Johansson *et al*, 2008). In prostate cancer patients this switch to Th2 polarisation occurs in association with development of metastatic disease (Filella *et al*, 2000), possibly explaining why the inverse association between *P. acnes* titres and prostate cancer risk would be stronger for advanced-stage disease.

Several lines of evidence support the possible involvement of immune responses against *P. acnes* in protection against prostate cancer. First, *P. acnes* is a potent immunomodulator that has been trialed as a cancer treatment in both animals and humans. The tumouricidal activity of *P. acnes* is mediated through recruitment and activation of innate immune cells including macrophages and natural killer T cells (Ananias *et al*, 2007). Second, acne has been indicated as a protective factor against various other disorders associated with a Th2 immune response, including melanomas, lymphomas, leukaemias, atopy, allergy, and asthma (Namazi, 2007). Finally, the inflammation commonly observed in the prostate gland is predominantly composed of Th1 lymphocytes and macrophages, which indicates a delayed-type hypersensitivity (Th1-type) immune response (McClinton *et al*, 1990; Sfanos *et al*, 2008).

As our study collected blood samples from adult men, we cannot distinguish between high titres established by acne during puberty and those arising later in response to prostate infection. Involvement of immunity levels maintained since puberty may be possible since previous research by Ingham *et al* (1987) has shown that antibody titres against *P. acnes* are established in early puberty and still exist at similar levels in individuals 65 years old. In that study the mean log_2_ titres were slightly higher than the ones we found in our case–control study, but the absolute values cannot be compared directly due to differences in assay sensitivity. However, since we observed that higher anti-*P. acnes* antibody titres were inversely associated with prostate cancer risk independently of a history of acne during adolescence, it is possible that in some men higher titres indicate infection of the prostate gland.

While this study supports our previous finding of an inverse association between facial acne scarring and prostate cancer risk in the RFPCS study (Giles *et al*, 2003), it is difficult to reconcile this observed inverse association with our previous finding that *P. acnes* culture-positive radical prostatectomy specimens were more likely to contain foci of acute or chronic inflammation (Cohen *et al*, 2005). In that study, however, it was not possible to investigate the correlation between the inflammatory immune response in the prostate with antibody levels in circulation because serum was not available.

In conclusion, the inverse association that we have observed between concentrations of circulating *P. acnes* antibodies and risk of prostate cancer needs to be replicated in large prospective studies to exclude the possibility that the increased concentrations in cases are caused by the tumour itself or by treatment. If our finding is confirmed it might open an avenue for the prevention of this disease.

## Figures and Tables

**Table 1:** Characteristics of participants in the Australian Risk Factors for Prostate Cancer Study

**Factor**	**Cases (%) *N*=809** a	**Controls (%) *N*=584** a
*Reference age*		
<55 years	113 (14)	111 (19)
55–59 years	194 (24)	98 (17)
60–69 years	502 (62)	375 (64)
		
*Country of birth*		
Australia	567 (70)	383 (66)
Overseas	241 (30)	200 (34)
		
*Self-reported history of acne in adolescence*		
No	641 (79)	456 (78)
Yes	167 (21)	125 (22)
		
*Facial acne scarring*		
Not obvious	735 (91)	518 (89)
Obvious	27 (3)	28 (5)
Uncertain (beard)	35 (4)	37 (6)

a The number of missing values for cases and controls were: 1 and 1 for country of birth; 1 and 3 for self-reported history of acne in adolescence; 12 and 1 for facial acne scarring.

**Table 2:** Odds ratios (OR) and 95% confidence intervals (95% CI)^a^ for prostate cancer for *P.acnes* antibody titres in the Risk Factors for Prostate Cancer Study

			**Overall**		**Tumour stage** b			**Tumour grade** c		
	**Cases**	**Controls**			**I–II (*N*=558)**	**III–IV (*N*=247)**		**Moderate (*N*=590)**	**High (*N*=219)**	
	***N***	***N***	**OR (95% CI)**	***P*-trend** d	**OR (95% CI)**	**OR (95% CI)**	***P*-trend** e	**OR (95% CI)**	**OR (95% CI)**	***P*-trend** e
*P. acnes titres (median)*										
<1 : 1024	402	252	Reference	0.005	Reference	Reference	0.07	Reference	Reference	0.8
⩾1 : 1024	407	332	0.73 (0.58–0.91)		0.79 (0.62–1.01)	0.59 (0.43–0.81)		0.72 (0.56–0.91)	0.75 (0.54–1.04)	
										
*P. acnes titres* (continuous)										
(Doubling concentration)	809	584	0.94 (0.89–0.99)	0.03	0.96 (0.90–1.01)	0.91 (0.85–0.98)	0.2	0.94 (0.89–0.99)	0.96 (0.89–1.03)	0.6
										
*P. acnes titres (tertiles)*										
1st tertile (<1 : 512)	229	159	Reference	0.06	Reference	Reference	0.6			0.7
2nd tertile (1 : 512– 1 : 2048)	303	195	1.00 (0.76–1.34)		1.00 (0.74–1.37)	1.00 (0.68–1.48)		1.03 (0.76–1.39)	0.93 (0.61–1.41)	
3rd tertile (>1 : 2048)	277	230	0.80 (0.60–1.06)		0.84 (0.62–1.14)	0.71 (0.48–1.05)		0.78 (0.58–1.06)	0.83 (0.55–1.25)	

a Estimates from logistic regression analyses adjusted for batch, reference age (<55, 55–59,60–69), study centre (Melbourne and Perth), selection year (1994, 1995, 1996, 1997).

b Four cases had missing stage.

c Moderate: Gleason score 5–7 or moderately differentiated tumours. High: Gleason score 8–10 or poorly differentiated or undifferentiated tumours.

d Likelihood ratio test. For the analysis by the median and by tertiles the test is based on the pseudo-continuous variable.

e Test for homogeneity of ORs across categories of tumour stage or grade.
